# Integrative analysis of blood biochemistry and sperm metabolome profiles reveals metabolic determinants of cryotolerance in Bali bulls under standardized nutritional conditions

**DOI:** 10.7717/peerj.21244

**Published:** 2026-05-20

**Authors:** Zulfi Nur Amrina Rosyada, Hikmayani Iskandar, Mohammad Anam Al Arif, Widya Paramita Lokapirnasari, Rimayanti, Siti Rani Ayuti, Herry Sonjaya, Erni Damayanti, Ananda Ananda, Hendri Hendri, Muhamad Aldi Nurdiansyah, Tulus Maulana, Erdogan Memili, Mirni Lamid

**Affiliations:** 1 Division of Animal Husbandry, Department of Veterinary Science, Faculty of Veterinary Medicine, Universitas Airlangga, Surabaya, East Java, Indonesia; 2 Animal Science and Nutrition Research Group, Faculty of Veterinary Medicine, Universitas Airlangga, Surabaya, East Java, Indonesia; 3 Research Center for Applied Zoology, National Research and Innovation Agency (BRIN), Bogor, West Java, Indonesia; 4 Division of Veterinary Reproduction, Department of Veterinary Science, Faculty of Veterinary Medicine, Universitas Airlangga, Surabaya, East Java, Indonesia; 5 Reproduction Engineering Research Group, Faculty of Veterinary Medicine, Universitas Airlangga, Surabaya, East Java, Indonesia; 6 Laboratory of Biochemistry, Faculty of Veterinary Medicine, Universitas Syiah Kuala, Kota Banda Aceh, Aceh, Indonesia; 7 Department of Animal Production, Faculty of Animal Science, Universitas Hasanuddin, Makassar, South Sulawesi, Indonesia; 8 Department of Animal Production Technology, Faculty of Animal Science, Universitas Andalas, Padang, West Sumatra, Indonesia; 9 Cooperative Agricultural Research Center, College of Agriculture, Food, and Natural Resources, Prairie View Agricultural and Mechanical University, Prairie View, TX, United States of America

**Keywords:** Bali bulls, Food security, GC-MS, Sperm metabolomics, Standardized diet

## Abstract

**Background:**

Enhancing the nation’s beef supply and guaranteeing long-term food security require increasing the effectiveness of artificial insemination (AI) in Bali cattle (*Bos javanicus*). Cryotolerance, or post-thaw variability in semen quality, limits AI results and probably reflects systemic and cellular metabolic variations. To determine the metabolic determinants of cryotolerance under standardized nutrition and supervision, this study combined blood biochemistry with the sperm metabolome.

**Methods:**

Ten healthy breeding bulls at the regional artificial insemination center (RAIC) were maintained on a uniform forage–concentrate diet. Blood biochemical parameters were measured using ethylenediaminetetraacetic acid (EDTA) plasma. For metabolomics, washed sperm pellets were prepared from frozen–thawed semen straws and profiled using untargeted gas chromatography–mass spectrometry (GC–MS). Multivariate analyses (hierarchical clustering, K-means, and partial least squares–discriminant analysis (PLS-DA) with variable importance in projection (VIP) scores) summarized the global patterns. Spearman correlations were used to integrate blood indices, sperm metabolites, and post-thaw traits (motility, viability, plasma membrane integrity (%PMI), and morphological abnormalities).

**Results:**

Eighteen intracellular metabolites were identified, predominated by fatty acyls. Unsupervised clustering and PLS-DA revealed clear inter-individual separation, with palmitic and stearic acids being among the most discriminant features (VIP ≥ 1.0). Systemic markers of lipid carriage and ionic tone aligned with sperm lipid composition: albumin and potassium were associated with higher intracellular palmitate levels and related metabolites. Functionally, lipid and short-chain fatty acid features, 1-monopalmitin, nonadecanoic acid, caproic acid, and valeric acid, were positively associated with viability and/or PMI%, whereas dodecanoic acid and glycerol monostearate were inversely related to morphological abnormalities. However, no robust association was detected with motility.

**Discussion:**

Under a controlled dietary baseline, a lipid-centric blood-to-sperm metabolic axis emerges as a key determinant of cryotolerance in *B. javanicus*. The prioritized metabolites constitute practical biomarker candidates for sire screening and provide a mechanistic basis for refining extenders and cryopreservation protocols at AI centers. Targeted tandem mass spectrometry, membrane-focused lipidomics, and mitochondrial functional assays offer immediate paths to translation, with the potential to improve reproductive efficiency and, ultimately, bolster sustainable beef production and food security.

## Introduction

Insufficient beef availability continues to pose a significant challenge in meeting Indonesia’s food demands. According to Badan Pusat Statistik (BPS) (2024), the national beef cattle population has fluctuated, starting at 16.43 million head in 2018, rising to 17.98 million head in 2021, then sharply declining to 10.83 million head in 2023, before increasing again to 11.75 million head in 2024. In 2024, beef and buffalo meat production is projected to total 496.25 thousand tons, reflecting a 3.93% increase from the previous year’s output of 477.46 thousand tons. The national output still falls short of demand (759.67 thousand tons), leaving an estimated deficit of 263.42 thousand tons. With per-capita beef intake projected at 2.8–3.0 kg per year (Ministry of Agriculture of the Republic of Indonesia, 2023), this supply–demand mismatch persists and poses ongoing risks to food security. One major but under-recognized contributor to this deficit is suboptimal reproductive efficiency in breeding bulls, particularly variability in semen quality after cryopreservation used for artificial insemination programs (Rosyada et al., 2023; Agil et al., 2025).

Despite improvements in herd management, reproductive inefficiency at the level of breeding bulls remains insufficiently characterized at the molecular level. In particular, the biological determinants underlying variability in sperm cryotolerance remain poorly defined, limiting the development of predictive biomarkers for artificial insemination success. Local breeds, especially Bali cattle (*B. javanicus*), are a key part of Indonesia’s herd as they fare well in tropical climates and are kept by many smallholders. National estimates show that Bali cattle comprise approximately 27% of the national herd (Purwantara, Handiwirawan & Noor, 2012). According to recent genetic data, this percentage has increased to approximately 32.3% (Jakaria et al., 2020). These figures demonstrate the significance of this breed for the long-term viability of domestic cattle production. In Bali cattle, artificial insemination (AI) is crucial for genetic dispersal; however, post-thaw sperm quality fluctuations severely impair AI efficacy. Bulls’ sperm viability and plasma membrane integrity can vary as a result of cryopreservation, which can cause oxidative stress, lipid phase transitions, and significant metabolic changes (Peris-Frau et al., 2020; Ananda et al., 2025). These differences indicate intrinsic metabolic and biochemical distinctiveness, frequently referred to as cryotolerance. Conventional sire selection mostly relies on macroscopic and microscopic semen properties; however, these measurements do not fully capture the molecular elements affecting sperm resilience during freezing and thawing. Consequently, the absence of predictive physiological or molecular biomarkers limits the ability of breeding centers to identify bulls with superior cryoresilience prior to semen processing.

Recent omics research has shed light on why certain spermatozoa are more tolerant of freezing than others. Large-scale proteomic work has catalogued more than 2,000 proteins in bovine sperm, including glycolytic and fatty acid–oxidation enzymes such as GAPDH, ACO2, and CPT1A, which support energy supply and antioxidant defense (Menezes et al., 2019; Talluri et al., 2022). These results were confirmed by metabolomic analysis, which identified more than 100 intracellular metabolites associated with fertility. For example, carnitine, lactic acid, and citric acid are often associated with motility and plasma membrane integrity (DasGupta et al., 2022; Pang et al., 2025). Proteome mapping in Indonesia has identified 15 tricarboxylic acid cycle enzymes in Madura bulls (Rosyada et al., 2023) and 94 seminal plasma proteins in Bali bulls (Iskandar et al., 2023), demonstrating the molecular diversity of the native breeds. However, no integrative study has simultaneously evaluated systemic metabolic indicators and sperm intracellular metabolome under standardized nutritional conditions to determine their combined contribution to cryotolerance in tropical indigenous bulls. This lack of integrative knowledge represents a critical scientific problem because sperm metabolic composition is shaped not only by intrinsic testicular processes but also by whole-body metabolic status. This creates a significant gap in our knowledge of how metabolic alterations affect cryotolerance.

The reproductive metabolic state of cattle is influenced by their diet. Dietary nutrients modulate circulating glucose, fatty acids, amino acids, and electrolytes, which determine systemic energy balance and redox status. Individuals differ in how they use nutrients, control hormones, and maintain redox balance, which leads to distinct metabolic profiles, even when given the same rations (Sammad et al., 2022). Basic blood chemistry, which includes the main electrolytes, glucose, total protein, and urea, provides a useful indicator of protein and energy turnover as well as systemic conditions. Nutritional inputs modulate circulating metabolites, endocrine signaling pathways, and systemic antioxidant capacity, thereby collectively influencing the biochemical conditions within reproductive organs (Agarwal et al., 2014; D’Occhio, Baruselli & Campanile, 2019). Circulating metabolites reach the testis through testicular microvasculature and influence Sertoli cell metabolism, while in the epididymis they contribute to luminal fluid composition that supports sperm maturation (Velho et al., 2018). Metabolites transported *via* blood can cross reproductive barriers and serve as substrates for mitochondrial respiration, membrane lipid remodeling, and osmotic regulation in developing spermatozoa, thereby influencing the intracellular metabolome that determines stress tolerance during freezing (Martin-Hidalgo et al., 2018). Systemic metabolism and cellular biochemistry during cryopreservation may be better understood by evaluating blood indices in conjunction with the sperm metabolome.

In this study, untargeted GC–MS was used to analyze Bali bull spermatozoa, and the data were merged with blood biochemical measurements to uncover metabolic variables linked with cryotolerance, including post-thaw motility, vitality, plasma membrane integrity, and morphological normality. At the regional artificial insemination center (RAIC) in Pucak, Maros, South Sulawesi, all bulls were provided the same feed and husbandry to ensure that biological differences were due to intrinsic physiology rather than nutrition. We hypothesized that specific blood biochemical indicators and sperm metabolites would correlate with superior post-thaw performance, reflecting intrinsic reproductive efficiency. If validated, such relationships could enable the development of minimally invasive biomarkers for selecting superior AI sires and for designing nutrition-based management strategies to enhance fertility outcomes. These interrelations will be mapped to identify bulls with stronger systemic and gametogenic metabolism under standard conditions and lay the groundwork for biomarker-based sire selection and nutrition-informed reproductive management to improve fertility and cryoresilience in indigenous cattle. Establishing these integrative metabolic relationships may enable early identification of high-cryotolerance bulls prior to semen freezing, reduce processing losses in AI centers, and ultimately improve reproductive efficiency and national beef production sustainability.

## Materials & Methods

### Ethical approval

All animal procedures were approved by the Animal Care and Use Committee (ACUC), Faculty of Veterinary Medicine, Universitas Airlangga (Ethical Clearance No. 1.KEH.107.07.2025, and were performed following the national guidelines for the Care and Use of Experimental Animals.

### Experimental animals and sample collection

This study was conducted using Bali bulls (*B. javanicus*) maintained at the Regional Artificial Insemination Center (RAIC) Pucak, Maros, South Sulawesi, Indonesia. Ten healthy, fertile Bali bulls aged 5–10 years were included in the study. The animals were maintained under standard husbandry conditions according to the institutional management guidelines of the RAIC, with routine health examinations and balanced feeding programs. All bulls had ≥70% fresh semen motility based on RAIC records. Semen for metabolomics was derived from archived frozen straws corresponding to ejaculates with available post-thaw quality records, allowing correlation of metabolites with phenotype without re-evaluation. Straws were thawed at 37 °C for 30 s, and the contents were transferred to sterile 15 mL conical tubes. Frozen semen was prepared using Andromed extender. Thus, the extender and cryoprotectant were removed by centrifugation at 3,000 × g for 30 min at 4 °C, followed by two washes with phosphate-buffered saline (PBS, pH 7.4) to minimize the carry-over of non-cellular components. The washed sperm pellets were aliquoted into cryogenic vials, snap-frozen in liquid nitrogen, and stored at −80 °C until metabolomic analysis. The handling times and temperatures were standardized across bulls to minimize preanalytical variation*.* All ten fertile Bali bulls available at the RAIC and meeting the semen quality criteria were included in this exploratory analysis; therefore, no a priori sample-size calculation was performed. Randomization was not performed as all bulls were maintained under identical conditions. Laboratory personnel performing semen processing and GC-MS analyses were aware of sample identity; however, quantification relied on automated instruments to minimise observer bias. Semen collection was followed standard operating procedures of the RAIC using minimal restraint to ensure animal welfare and to reduce handling-related stress. Blood and semen samples were collected within the same sampling period for each animal under standardized feeding and management conditions to minimize temporal biological variation between systemic biochemical measurements and sperm metabolomic profiles.

### Feed sampling and analysis

Feed samples were collected from the RAIC Pucak, Maros, South Sulawesi facility, which provides a standardized diet for all bulls. The bulls received a diet consisting of fresh forage (10–12% of body weight) and concentrate (1–2% of body weight), offered twice daily at 06:30 and 16:00 h. During the study period, representative mixed-feed samples were collected and analyzed in triplicate using Near-Infrared Reflectance Spectroscopy (NIRS™, FOSS DS2500) to determine the dry matter (DM), crude protein (CP), ether extract (EE), crude fiber (CF), and total ash. Analyses were performed following the AOAC (2019) calibration standards. The feed composition data were used to confirm that all bulls received a uniform and nutritionally adequate diet. These values served as background nutritional information but were not included in subsequent correlation analysis.

### Blood collection and examination of biochemical profiles

Approximately three mL of blood was drawn from each bull’s jugular vein using sterile syringes and vacutainer tubes coated with EDTA. At ∼10:30 h, 3–4 h post-feeding, to minimize postprandial effects. The plasma was separated by centrifugation at 1,006 × g for 15 min at 4 °C and it was then kept for later analysis at −20 °C. Using a kidney profile analyser (IDEXX Laboratories), biochemical components such as total protein (TP), albumin, globulin, glucose, blood urea nitrogen (BUN), creatinine, calcium (Ca^2^^+^), inorganic phosphate (Pi), sodium (Na^+^), potassium (K), chloride (Cl^−^), and total carbon dioxide (TCO_2_) were measured in compliance with the manufacturer’s instructions. These variables were considered markers of nutritional uptake and metabolic efficiency in each animal. The data were coupled with sperm metabolomic profiles for further study and are expressed in g/dL, mg/dL, or mmol/L, depending on the parameter. Normality was assessed using the Shapiro–Wilk test. Normal distribution variables were expressed as mean ± SD, and non-normally distributed values were expressed as median (IQR).

### Post-thaw sperm motility evaluation

Post-thaw sperm quality parameters, including motility, viability, plasma membrane integrity, and morphological abnormalities, were evaluated using the same semen batches used for metabolomics. Frozen semen was thawed at 37 °C for 30 s, and sperm motility was assessed using a computer-assisted sperm analysis (CASA) (SpermVision, Minitüb, Germany) system. Viability, plasma membrane integrity, and morphological abnormalities were evaluated using eosin–nigrosin staining and hypo-osmotic swelling test, consistent with standard bovine sperm evaluation protocols (Rosyada et al., 2021). These post-thaw sperm quality parameters, obtained from existing RAIC records, were used exclusively as phenotypic variables for correlation with sperm metabolite abundance and were not reanalysed in this study.

### Spermatozoa analysis using gas chromatography-mass spectrometry (GC-MS)

Prior to GC–MS analysis, intracellular metabolites were extracted from sperm pellets following a modified protocol based on Fiehn (2017), Velho et al. (2018), and Menezes et al. (2019). Frozen semen straws were thawed at 37 °C for 30 s and centrifuged at 4,730× g for 30 min at 4 °C to separate cellular and extracellular fractions. After centrifugation, the supernatant was discarded and the sperm pellet was retained for metabolite extraction. The pellet was mixed with 150 μL heptadecanoic acid in methanol (one mg/mL; internal standard) and 350 μL extraction solution (ultrapure water:methanol, 1:4 v/v), vortexed for 1 min, and centrifuged at 12,000 × g for 10 min at 4 °C. The resulting supernatant was filtered through a syringe filter and transferred to a clean tube. Solvents were evaporated to dryness under a gentle nitrogen stream at 36 °C for approximately 2 h using a TurboVap^^®^^ LV evaporator (Biotage). Dried extracts were derivatized by adding 50 μL methoxyamine hydrochloride (20 mg/mL in pyridine), vortexing for 1 min, and incubating at 30 °C for 1 h, followed by addition of 100 μL N-trimethylsilyl-N-methyl trifluoroacetamide containing 1% trimethylchlorosilane (MSTFA + 1% TMCS) and incubation at 70 °C for 1 h. Samples were centrifuged at 13,000× g for 10 min at 4 °C, and the supernatant was transferred to two mL amber GC vials with inserts. Two pooled quality-control samples were prepared by combining equal aliquots from all extracts and analyzed periodically to monitor analytical stability.

The metabolites of Bali bull spermatozoa were analyzed using a gas chromatography–mass spectrometry (GC–MS) system (GC-MS-QP2010, Shimadzu Corporation, Japan) equipped with an Rtx-5MS capillary column (30 m × 0.25 mm, 0.25 µm film thickness; Agilent Technologies, USA). A one μL aliquot of each liquid sample was injected into the GC–MS. The injector, interface, and ion source temperatures were set to 230, 250, and 200 °C, respectively. Helium (99.9% purity) was used as the carrier gas at a constant flow rate of three mL/min. The oven temperature program was as follows: initially maintained at 60 °C for 2 min, then increased to 300 °C at a rate of 15 °C/min, and held for 20 min. The solvent-cut time was set to 5 min. The mass spectrometer was operated in electron ionization (EI) mode at 70 eV, with a mass scan range of m/z 30–600. Compound identification was performed by matching the acquired mass spectra with the NIST 20 library and retention index data (https://webbook.nist.gov/chemistry/) using MS-Search software version 3.0 with the NIST 20 database (Wallace & Moorthy, 2023). Metabolites were identified based on their retention times and by comparing one target ion and two qualifier ions with those of authentic standards and spectra from the NIST library. The identified compounds were further categorized into chemical classes using the Human Metabolome Database (HMDB) version 5.0 classification ontology (https://www.hmdb.ca; Wishart et al., 2022).

### Metabolomic data processing and profiling of Bali bull spermatozoa

Metabolite peak intensity data obtained from GC–MS analysis were processed and analyzed using MetaboAnalyst 6.0 (https://www.metaboanalyst.ca/) for metabolic profiling. To avoid sample size inflation, three technical replicates from one representative sample were averaged prior to unsupervised clustering and partial least squares discriminant analysis (PLS-DA) modelling. A hierarchical heatmap was generated to visualize the global metabolite distribution patterns among the samples. Hierarchical clustering was performed using Ward’s minimum variance linkage (Ward.D2) with Euclidean distance for sample clustering and Pearson’s correlation for metabolite clustering (Sun et al., 2023; Pang et al., 2024).

K-means clustering was applied to identify groups of metabolites exhibiting similar abundance patterns. The optimal number of clusters was determined based on the elbow and silhouette criteria. Pearson’s correlation analysis was conducted to examine the associations among metabolites and between metabolites and sperm quality parameters. Correlation coefficients (r) were calculated using the Pearson method, and statistical significance was determined after false discovery rate (FDR) correction. Correlations with *r* ≥ 0.8 and FDR < 0.05 were considered to be significant. To identify discriminant metabolites between the sample groups, partial least squares discriminant analysis (PLS–DA) was performed as an exploratory supervised analysis to visualize metabolite patterns between sample groups. Model performance was assessed using 10-fold cross-validation. The metabolites that contributed most to group separation were identified based on variable importance in projection (VIP) scores, with features having VIP ≥ 1.0 considered the most influential. PLS–DA score plots and biplots were used to visualize the sample separation and metabolite contributions (Thanuma et al., 2025).

### Statistical analysis and integrative correlation study

All statistical analyses were performed using IBM SPSS Statistics v29.0 (IBM Corp., Armonk, NY, USA) and MetaboAnalyst 6.0 (https://www.metaboanalyst.ca/). Feed and blood data were analyzed descriptively to confirm uniform dietary composition and characterize individual systemic metabolic variations among bulls. The feed composition parameters (DM, CP, EE, CF, and total ash) were determined from mixed feed samples (forage–concentrate), analyzed in triplicate, and expressed as mean ± standard deviation (SD). Blood biochemical parameters were summarized using descriptive statistics. Normality was tested using the Shapiro–Wilk test, and the results were reported as mean ± SD for normally distributed variables or median (IQR) for non-normal variables. Feed composition values were not included in the correlation analyses because the feeding regime was intentionally standardized across all bulls; they served as a background for interpreting systemic and sperm metabolic profiles and therefore lacked between-animal variability required for meaningful correlation testing. Integrative correlation analyses were conducted to examine the associations between blood biochemistry, sperm metabolite abundances (from GC–MS), and post-thaw sperm quality traits. Spearman’s rank correlation was used for these blood–sperm and sperm–phenotype associations. Correlation coefficients (r_s_) and two-tailed *P-values* were calculated, with statistical significance defined as *P* < 0.05. No multiple testing correction was applied; results were interpreted as hypothesis-generating, and r_s_ ≥ 0.8 was considered biologically strong. To reduce the likelihood of spurious associations, this stringent effect-size threshold was applied in addition to statistical significance criteria. Multivariate analyses (hierarchical clustering, k-means, and PLS-DA) were validated as described above, prior to integrating the metabolomic, biochemical, and phenotypic layers. Because the present dataset comprised experimentally detected metabolites rather than comprehensive metabolome coverage, interpretation was performed at the individual metabolite level, and no pathway enrichment analysis was conducted.

## Results

### Feed composition

Feed composition analysis by near-infrared reflectance spectroscopy (NIRS) revealed that the mixed diet (fresh forage and concentrate) provided at RAIC Pucak, Maros, exhibited consistent nutritional quality across biological replicates (Table 1). The mixed diet contained, 89.16 ± 0.13% dry matter, 10.42 ± 1.07% crude protein, 4.29 ± 0.49% ether extract, 31.08 ± 0.88% crude fiber, and 9.80 ± 0.24% total ash. These nutrient levels were within the expected range for breeding bulls (CP 10–12%; CF > 20%) according to the RAIC Pucak, Maros, South Sulawesi, Indonesia feeding standards, suggesting that the ration provided adequate protein and fiber to support normal metabolic and reproductive functions. Minor variation among replicates indicated a stable diet formulation. Feed data were therefore used descriptively as background nutritional context and were not included in correlation analyses.

**Table 1:** Nutrient composition of mixed feed samples (forage and concentrate) provided to Bali bulls (mean ± SD).

Parameter	Mean ± SD (% DM basis)
Dry Matter	89.16 ± 0.13
Crude Protein	10.42 ± 1.07
Ether Extract	4.29 ± 0.49
Crude Fiber	31.08 ± 0.88
Total Ash	9.80 ± 0.24

**Notes.**

Values are mean ± SD of six mixed-feed samples analyzed in triplicate by NIRS). DM: Dry Matter.

### Blood biochemical profile of Bali bulls

The blood biochemical indicators of the ten Bali bulls were presented in Table 2. The central tendency and dispersion were reported as mean ± standard deviation (SD) for normally distributed variables and median (interquartile range, IQR) for non-normally distributed variables. The values were: total protein (TP) 7.54 ± 0.70 g/dL, albumin 2.92 ± 0.25 g/dL, globulin 4.62 ± 0.57 g/dL (unit corrected), glucose 48.3 ± 5.17 mg/dL, creatinine 2.61 ± 0.18 mg/dL, and BUN 11.9 (10.0–13.5) mg/dL (median, IQR). The electrolyte and mineral concentrations were as follows: Na^+^ 138.4 ± 4.17 mmol/L, K^+^ 5.08 (4.55–5.77) mmol/L (median, IQR), Cl^−^ 98.3 ± 3.59 mmol/L, TCO_2_ 26.2 ± 1.87 mmol/L, Ca^2^^+^ 9.40 ± 0.38 mg/dL, and Pi 3.98 ± 1.13 mg/dL. Shapiro–Wilk tests indicated non-normal distributions for TP (*P* = 0.036), BUN (*P* = 0.009), and K^+^ (*P* = 0.046); all other variables were normal (*P* > 0.05) and were thus summarized as mean ± SD. No statistical outliers were detected based on box-and-whisker inspection and IQR criteria, confirming the homogeneity of the systemic biochemical data across bulls. Descriptive statistics of these biochemical variables were subsequently used for correlation analyses with the sperm metabolite profiles.

**Table 2:** Blood biochemical parameters of Bali bulls.

Parameter	Mean ± SD/Median (IQR)	Normality (Shapiro–Wilk *P)*
TP (g/dL)	7.54 ± 0.70	0.036^*^
Albumin (g/dL	2.92 ± 0.25	0.083
Globulin (g/dL)	4.62 ± 0.57	0.100
Glucose (mg/dL)	48.3 ± 5.17	0.341
Creatinine (mg/dL)	2.61 ± 0.18	0.246
BUN (mg/dL)	11.9 (10.0–13.5)	0.009^*^
Ca^2^^+^(mg/dL)	9.40 ± 0.38	0.590
Pi (mg/dL)	3.98 ± 1.13	0.872
Na^+^ (mmol/L)	138.4 ± 4.17	0.933
K^+^ (mmol/L)	5.08 (4.55–5.77)	0.046^*^
Cl^−^ (mmol/L)	98.3 ± 3.59	0.916
TCO_2_ (mmol/L)	26.2 ± 1.87	0.233

**Notes.**

Data are expressed as mean ± SD for normally distributed variables, and median (IQR) for non-normally distributed ones.

* Non-normal distribution (*P* < 0.05).

Total protein (TP); Blood urea nitrogen (BUN); inorganic phosphate (Pi); sodium (Na^+^), potassium (K), chloride (Cl^−^), and total carbon dioxide (TCO_2_).

### Metabolites identification and profiling of Bali bull spermatozoa

A total of 18 organic metabolites were identified in the spermatozoa of 10 Bali bulls using GC–MS. The metabolites were categorized into eight chemical classes and presented numerically to allow precise comparison of proportional distribution. Fatty acyls represented the largest chemical class in terms of metabolite count (38.9%); however, based on relative abundance, fatty acyls dominated the sperm metabolome (67.67%). This was followed by pyridinecarboxylic acids (19.76%), carboxylic acids and derivatives (4.22%), and steroids and steroid derivatives (3.86%). Minor classes included organooxygen compounds (1.64%), hydroxy acids and derivatives (1.37%), glycerolipids (0.79%), and organonitrogen compounds (0.70%). This distribution highlights the predominance of lipid-related metabolites, suggesting their potential role in membrane stability and sperm cryotolerance. Among the identified metabolites, palmitic acid demonstrated the greatest relative abundance, followed by stearic acid, 6-methyl-2-pyridinecarboxylic acid, and valeric acid (Table 3). Additionally, 11 metabolites were uniformly identified in all bulls, including 6-methyl-2-pyridinecarboxylic acid, valeric acid, lactic acid, caproic acid, ethanolamine, citric acid, myristic acid, D-fructose, palmitic acid, stearic acid, and nonadecanoic acid. Serine was identified only in the Rowa bull sample, indicating potential individual-specific metabolic variance.

**Table 3:** Metabolites identified in Bali bull spermatozoa by GC–MS and their relative abundances. Sperm metabolites detected in Bali bull semen using GC–MS. Compounds are grouped by chemical class and ordered by retention time (RT). Values represent the relative peak area (%) for each bull; “nt” indicates not trace (peak below the detection threshold).

Chemical class (Mean %)	Compound name	RT	Peak area (%)									
			Arjuna	Dewa	Hercules	Kajuara	Lewa	Maiwa	Rewa	Rowa	Singo	Sinyo
Fatty acyls (67.67%)	Valeric acid	5.16	13.78	15.53	15.51	14.48	16.65	13.03	16.53	14.89	15.49	15.29
	Caproic acid	6.28	0.81	0.88	1.13	1.03	1.21	0.68	1.01	0.86	0.98	0.74
	Dodecanoic acid	11.66	0.42	0.64	nt	0.44	0.44	0.46	0.42	0.38	0.56	0.61
	Myristic acid	13.08	1.48	1.73	1.35	1.37	1.13	1.54	1.27	1.60	1.25	1.59
	Palmitic acid	14.38	25.93	26.23	27.39	27.34	27.64	24.54	24.52	31.71	25.94	31.46
	Stearic acid	15.58	21.33	19.63	21.45	22.15	21.16	21.28	20.06	24.12	19.85	23.32
	Nonadecanoic acid	16.14	1.21	0.96	1.19	1.06	0.96	0.85	1.00	0.78	1.28	1.13
Pyridinecarboxylic acids and derivatives (19.76%)	6-Methyl-2- pyridinecarboxylic acid	5.08	18.98	19.32	20.03	19.84	22.25	16.85	21.30	16.51	22.74	19.72
Carboxylic acids and derivatives (4.22%)	Glycine	8.80	1.49	3.67	1.59	2.52	nt	2.14	2.33	1.41	1.84	1.10
	Serine	9.29	nt	nt	nt	nt	nt	nt	nt	0.65	nt	nt
	Citric acid	12.99	2.58	4.11	1.64	2.47	0.84	4.30	2.47	2.01	2.31	0.71
Steroids and steroid derivatives (3.86%)	Calcitriol	15.48	0.42	1.38	nt	0.76	1.94	nt	nt	nt	1.06	0.78
	Cholesterol	21.33	7.74	nt	3.04	1.95	1.89	8.13	3.22	2.04	3.21	1.03
Organooxygen compounds (1.64%)	D-Fructose	13.42	1.83	2.82	1.15	1.73	0.76	2.96	1.84	1.02	1.68	0.59
Hydroxy acids and derivatives (1.37%)	Lactic acid	6.18	1.35	1.21	1.86	1.59	1.04	1.65	1.27	1.03	1.34	1.35
Glycerolipids (0.79%)	1-monopalmitin	17.47	nt	0.62	1.09	0.74	0.70	0.53	0.75	nt	nt	nt
	Glycerol monostearate	18.44	nt	0.71	0.92	nt	0.69	0.65	nt	0.47	nt	nt
Organonitrogen compounds (0.70%)	Ethanolamine	8.06	0.62	0.55	0.64	0.51	0.71	0.41	1.99	0.50	0.47	0.57

**Notes.**

Data are grouped by chemical class to facilitate the interpretation of metabolite distribution. The percentage contribution of each class represents the mean relative abundance across all bulls. nt, not detected; RT, retention time.

**Figure 1:**
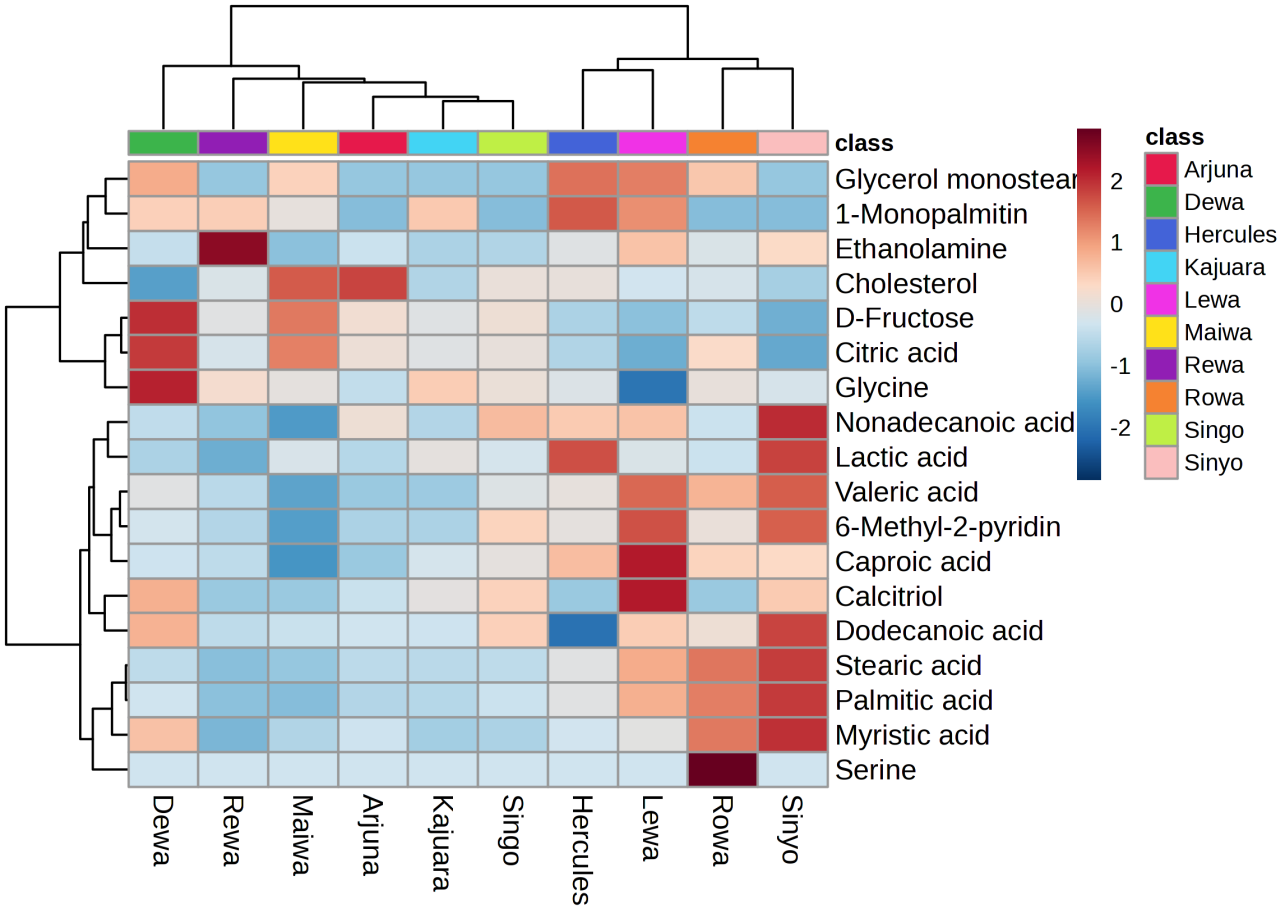
Heatmap of normalized spermatozoa metabolite profiles in Bali bulls. Columns represent individual bulls and rows represent metabolites. Colors indicate relative abundance (z-score), where darker blue denotes lower relative abundance and darker red denotes higher relative abundance.

**Figure 2:**
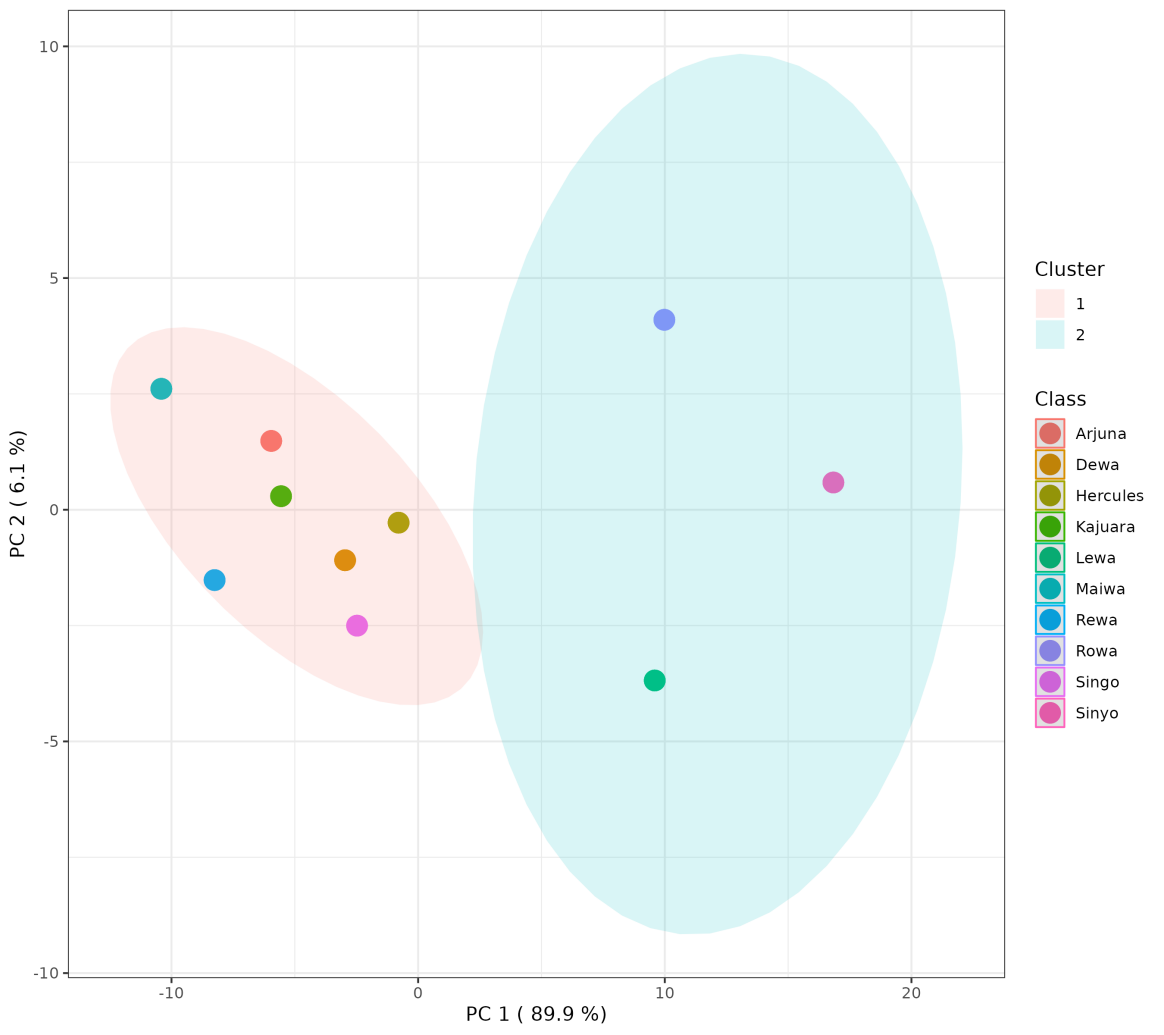
K-means clustering of Bali bull spermatozoa metabolite profiles. Principal component plot (PC1 89.9% and PC2 6.1%) showing K-means clustering of sperm metabolite profiles from Bali bulls. Each point represents one bull, with colors indicating individual bulls, and the shaded ellipses representing the two K-means clusters.

The identified metabolites were subjected to metabolic profiling using MetaboAnalyst. The heatmap visualization derived from the peak intensities indicated the presence of two distinct clusters. Cluster 1 consisted of Arjuna, Dewa, Kajuara, Maiwa, Rewa, and Singo, whereas Cluster 2 encompassed Hercules, Lewa, Rowa, and Sinyo (Fig. 1). K-means clustering yielded groupings: Cluster 1 included Arjuna, Dewa, Hercules, Kajuara, Maiwa, Rewa, and Singo, while Cluster 2 consisted of Lewa, Rowa, and Sinyo (Fig. 2). This concordance between clustering approaches indicates individual variability in the sperm metabolome despite standardized husbandry and nutrition.

**Figure 3:**
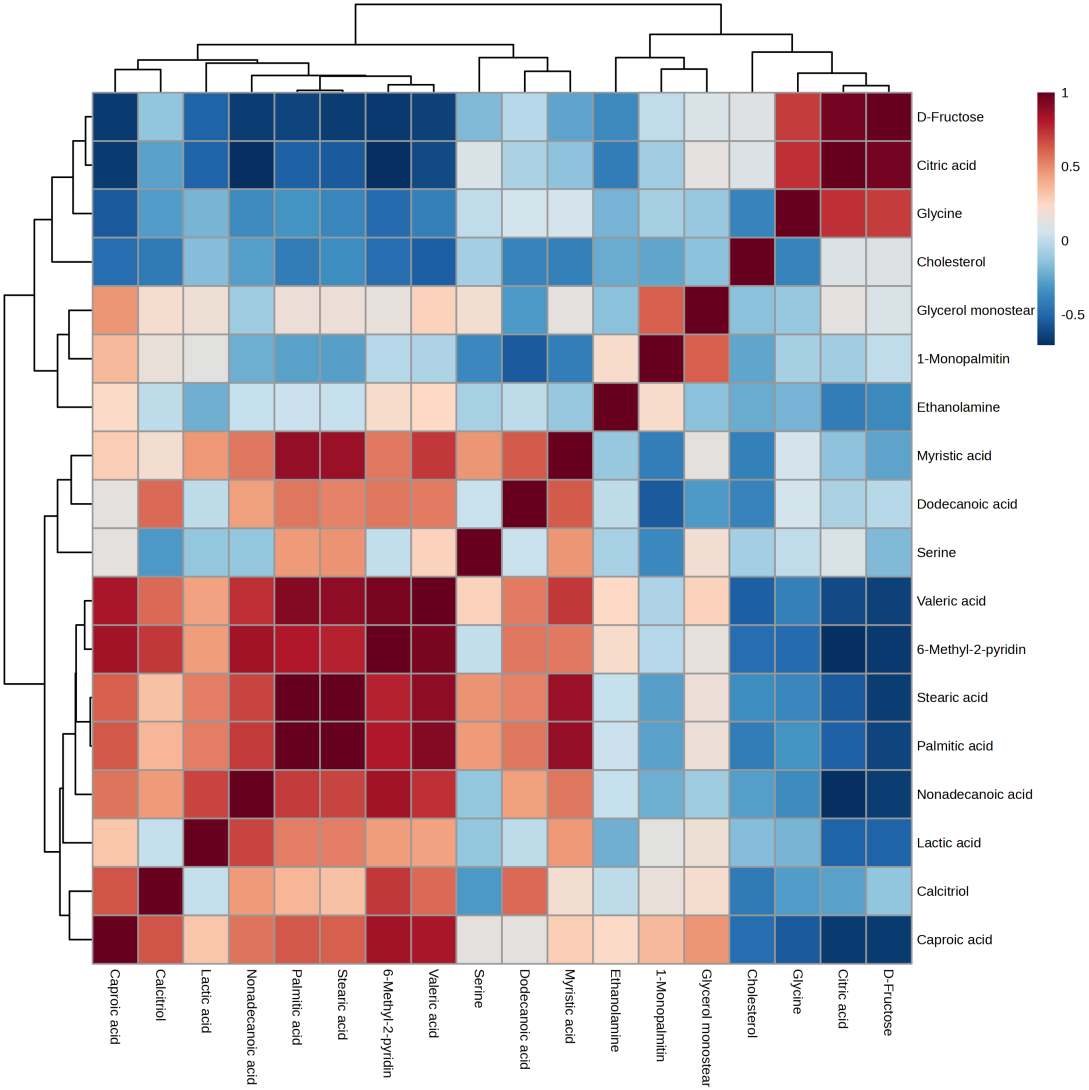
Heatmap of Pearson correlations among sperm metabolites in Bali bulls. Colors represent the strength and direction of the correlation (r), with deeper blue indicating stronger negative correlations and deeper red indicating stronger positive correlations (scale from –1 to +1).

Among the identified metabolites, Pearson correlation analysis revealed 11 strong positive relationships that persisted following multiple testing correction (FDR < 0.05, *r* > 0.8) (Fig. 3). D-fructose and citric acid (*r* = 0.96), palmitic acid and myristic acid (*r* = 0.88), stearic acid and myristic acid (*r* = 0.87), and stearic acid and palmitic acid (*r* = 0.99) were strongly correlated. Furthermore, there were significant positive correlations between valeric acid and stearic acid (*r* = 0.90) and palmitic acid (*r* = 0.92). Significant correlations were also observed for 6-methyl-2-pyridinecarboxylic acid with nonadecanoic acid (*r* = 0.85), palmitic acid (*r* = 0.81), and valeric acid (*r* = 0.95). In addition, there were positive relationships between caproic acid and 6-methyl-2-pyridinecarboxylic acid (*r* = 0.84) and valeric acid (*r* = 0.83).

**Figure 4:**
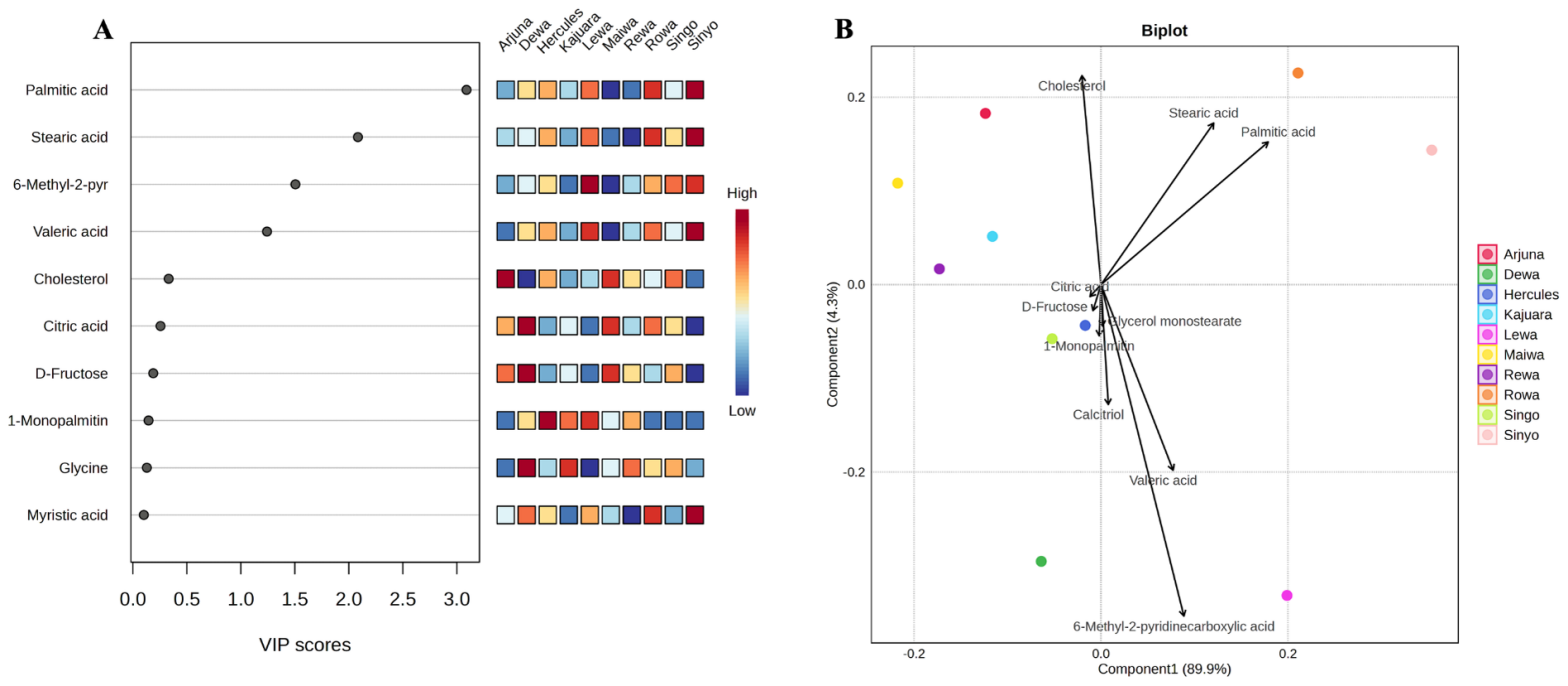
PLS-DA variable importance and biplot of key sperm metabolites in Bali bulls. (A) Variable importance in projection (VIP) scores for the 10 most influential metabolites, with the adjacent heatmap showing their relative abundance across individual bulls (darker blue = lower relative abundance, darker red = higher relative abundance). (B) PLS-DA biplot of the same 10 metabolites, where points represent individual bulls and arrows represent metabolite loadings on the first two components.

PLS-DA was conducted to differentiate metabolite patterns among bulls, accompanied by VIP scoring and biplot visualization to identify features that significantly contributed to sample separation. Based on VIP scores (≥1.0), four metabolites were identified as the primary contributors to group discrimination (Fig. 4A): palmitic acid (VIP = 3.09), stearic acid (VIP = 2.08), 6-methyl-2-pyridinecarboxylic acid (VIP = 1.51), and valeric acid (VIP = 1.24). The PLS-DA biplot (Fig. 4B) showed distinct separation among individuals, with increased concentrations of specific metabolites in Lewa, Rowa, and Sinyo bulls.

### Association between sperm metabolites and blood biochemical parameters

Spearman’s rank correlation analysis revealed multiple significant relationships between blood biochemical markers and sperm metabolites in Bali bulls (Table 4). There was a negative association between total protein (TP) and cholesterol (*r*_*s*_ = −0.787, *P* = 0.012) and a positive correlation with palmitic acid (*r*_*s*_ = 0.654, *P* = 0.04). Palmitic acid and albumin levels were positively correlated (*r*_*s*_ = 0.719, *P* = 0.019). Glycine and glucose showed a substantial negative correlation (*r*_*s*_ = −0.857, *P* = 0.003. A positive correlation was observed between 1-monopalmitin and blood urea nitrogen (BUN) levels (*r*_*s*_ = 0.883, *P* = 0.020). A negative association between calcium (Ca) and calcitriol (*r*_*s*_ = −0.886, *P* = 0.019). Potassium (K^+^) showed a mixed pattern of association; it was positively correlated with palmitic acid (*r*_*s*_ = 0.793, *P* = 0.006) and negatively correlated with citric acid (*r*_*s*_ = −0.688, *P* = 0.028) and D-fructose (*r*_*s*_ = −0.683, *P* = 0.030). K^+^ and cholesterol levels were inversely correlated (*r*_*s*_ = −0.740, *P* = 0.023). Total CO_2_ (TCO_2_) was negatively associated with glycerol monostearate (*r*_*s*_ = −0.894, *P* = 0.041), whereas chloride (Cl^−^) was positively associated with caproic acid (*r*_*s*_ = 0.716, *P* = 0.020). Other blood–sperm parameter pairs showed no significant correlations.

**Table 4:** Correlations between blood biochemical parameters and sperm metabolites in Bali bulls.

Blood biochemical indicators	Sperm metabolite	r_s_	*P*-value
TP (g/dL)	Palmitic acid	0.654^*^	0.04
	Cholesterol	−0.787^*^	0.012
Albumin (g/dL)	Palmitic acid	0.719^*^	0.019
Glucose (mg/dL)	Glycine	−0.857^**^	0.003
BUN (mg/dL)	1-Monopalmitin	0.883^*^	0.020
Ca (mg/dL)	Calcitriol	−0.886^*^	0.019
K+ (mmol/L)	Citric acid	−0.688^*^	0.028
	D-Fructose	−0.683^*^	0.030
	Palmitic acid	0.793^**^	0.006
	Cholesterol	−0.740^*^	0.023
Cl- (mmol/L)	Caproic acid	0.716^*^	0.020
TCO2 (mmol/L)	Glycerol monostearate	−0.894^*^	0.041

**Notes.**

Values represent Spearman’s rank correlation coefficients (r_s_).

* *P* < 0.05.

** *P* < 0.001.

Positive and negative values indicate positive and inverse associations, respectively. Total protein (TP); Blood urea nitrogen (BUN); inorganic phosphate (Pi); sodium (Na^+^), potassium (K), chloride (Cl^−^), and total carbon dioxide (TCO_2_).

### Association between post-thaw sperm quality parameters and metabolite abundance

The association between the amount of sperm metabolites and post-thaw sperm quality markers was presented in Table 5. Sperm Viability showed positive correlations with nonadecanoic acid (*r*_*s*_ = 0.423, *P* < 0.05) and 1-monopalmitin (*r*_*s*_ = 0.387, *P* < 0.05), and a negative correlation with calcitriol (*r*_*s*_ = −0.403, *P* < 0.05). Plasma membrane integrity (%PMI) correlated positively with caproic acid (*r*_*s*_ = 0.405, *P* < 0.01), valeric acid (*r*_*s*_ = 0.363, *P* < 0.01), 6-methyl-2-pyridinecarboxylic acid (*r*_*s*_ = 0.338, *P* < 0.01), 1-monopalmitin (*r*_*s*_ = 0.401, *P* < 0.05), and glycerol monostearate (*r*_*s*_ = 0.623, *P* < 0.01), and negatively with stearic acid (*r*_*s*_ = −0.339, *P* < 0.01). Morphological abnormalities were negatively correlated with glycerol monostearate (*r*_*s*_ = −0.697, *P* < 0.01), dodecanoic acid (*r*_*s*_ = −0.531, *P* < 0.01), nonadecanoic acid (*r*_*s*_ = −0.425, *P* < 0.01), glycine (*r*_*s*_ = −0.358, *P* < 0.01), and lactic acid (*r*_*s*_ = −0.312, *P* < 0.05). No significant correlations were detected between metabolite abundance and sperm motility (*P* > 0.05 for all comparisons) indicating no measurable association within the detected metabolite set.

**Table 5:** Correlation between post-thaw sperm quality parameters and spermatozoa metabolites in Bali bulls.

Variables (sperm metabolites and quality parameters)	Motility	Viability	Morphological abnormalities	Plasma membrane integrity (%PMI)
6-Methyl-2-pyridinecarboxylic acid	0.008	0.112	−0.126	**0.338** ^**^
Valeric acid	0.002	−0.038	0.132	**0.363** ^**^
Lactic acid	0.029	−0.254	−0.312^*^	−0.166
Caproic acid	0.014	−0.056	−0.238	**0.405** ^**^
Ethanolamine	0.035	0.039	0.209	0.143
Glycine	−0.023	−0.168	−0.358^**^	0.261
Dodecanoic acid	−0.010	0.014	−0.531^**^	0.222
Citric acid	−0.067	−0.213	0.029	0.059
Myristic acid	0.035	−0.036	−0.174	−0.067
D-Fructose	−0.017	−0.066	0.099	0.140
Palmitic acid	0.023	−0.079	−0.200	−0.107
Calcitriol	0.010	−0.403^*^	0.347	0.157
Stearic acid	0.023	0.024	−0.208	−0.339^**^
Nonadecanoic acid	0.009	**0.423** ^*^	−0.425^**^	0.252
1-Monopalmitin	0.069	**0.387** ^*^	−0.244	**0.401** ^*^
Glycerol monostearate	0.047	0.060	−0.697^**^	**0.623** ^**^
Cholesterol	0.041	0.075	0.260	−0.042
**Correlation among sperm quality parameters**				
Motility	1.000	0.065	−0.038	−0.133
Viability		1.000	−0.202	0.061
Abnormality			1.000	−0.202
Plasma integrity membrane (%PMI)				1.000

**Notes.**

Values represent Spearman’s rank correlation coefficients (r_s_).

* *P* < 0.05.

** *P* < 0.01.

Positive and negative values indicate positive and inverse associations, respectively. The lower section presents correlations among sperm quality parameters for reference.

The bold values indicate statistically significant correlations (*P < 0.05; **P < 0.01).

## Discussion

Feed analysis showed that the ration given to all Bali bulls at the RAIC had a stable nutrient profile and met the nutritional range recommended for breeding animals. On average, the diet contained 89.16 ± 0.13% dry matter, 10.42 ± 1.07% crude protein, and 31.08 ± 0.88% crude fiber. These values are comparable to those of previous reports on tropical beef and breeding bulls maintained on balanced forage–concentrate diets, which generally contain approximately 88–89% dry matter, 9.7–12% crude protein, and 10–29% crude fiber (Luthfi, Restitrisnani & Umar, 2018; Cagle et al., 2020; Kahyani et al., 2022). The slightly higher fiber level observed here most likely reflects the use of fresh forage as the main feed source at RAIC, Pucak, Maros, and South Sulawesi, Indonesia. Such a composition is favourable for Bali bulls because adequate fiber intake helps stabilize rumen function, reduces the risk of acidosis, and maintains a long-term metabolic balance (Allen, 1997). The small variation among samples indicates that the ration was consistently formulated throughout the study. Because all bulls were maintained on the same standardized diet, feed composition data were treated descriptively rather than as variables in the correlation analysis. This dietary uniformity provides a reliable baseline for interpreting the subsequent differences in the blood and sperm metabolic profiles of individuals.

Correspondingly, blood biochemical parameters provide a direct reflection of the systemic metabolic status, serving as a physiological bridge between the nutritional background and sperm intracellular metabolism. The mean concentrations of total protein (7.54 ± 0.70 g/dL), albumin (2.92 ± 0.25 g/dL), glucose (48.3 ± 5.17 mg/dL), and BUN (11.9 mg/dL, IQR = 10.0–13.5) were within the physiological ranges previously reported for tropical breeding bulls (TP: 7.10−6.81 g/dL; albumin: 2,67−4.24 g/dL; glucose: 43.00–53.98 mg/dL; BUN: 22.71–25.05 mg/dL), indicating adequate protein turnover and energy balance (Barson et al., 2019; Yang et al., 2022). Notably, BUN values tended toward the lower end of published reference intervals, further supporting the metabolic stability of the study population. These values confirm the absence of systemic metabolic imbalance, supporting stable physiological conditions across individuals maintained under standardized feeding and management conditions. Moreover, together with normal electrolyte and mineral levels (Na^+^, K^+^, Ca, and P), indicating a well-regulated systemic metabolism and efficient nutrient utilization across all individuals. The relative homogeneity of blood chemistry further supports that the observed differences in sperm metabolomic composition are unlikely to be diet-driven but rather reflect individual metabolic responsiveness and downstream effects on spermatogenic or epididymal processes.

The 18 metabolites found in Bali bull spermatozoa were categorized into eight chemical groups. Fatty acyls represented the largest chemical class in terms of metabolite count (38.9%); however, based on relative abundance, fatty acyls dominated the sperm metabolome (67.67%). This abundance-based dominance indicates that lipid-derived metabolites contribute substantially to the biochemical composition of spermatozoa, exceeding what is suggested by metabolite count alone. According to previous metabolomic studies in *B. taurus* and *B. indicus*, where lipid-derived compounds such as palmitic and stearic acids were also common, this pattern is consistent with those findings (Velho et al., 2018; Menezes et al., 2019). The proportion of fatty acyls in this study was marginally higher than the 22–25% reported by Menezes et al. (2019). The proportion of fatty acyls in this study appears higher than the 22–25% reported by Menezes et al. (2019), which may be attributed to differences in quantification approaches, particularly the use of relative peak area-based abundance rather than normalized metabolite ratios. Several studies have highlighted lipid-associated metabolites as key components in maintaining sperm membrane integrity and resistance to cryogenic stress (Peris-Frau et al., 2020). In this context, the predominance of fatty acyls observed in the present study reinforces the importance of lipid metabolism in supporting membrane stability and cryotolerance.

In addition to the compositional overview, multivariate analysis provided further evidence of metabolic variability among individual bulls. Fatty acyls, especially long-chain saturated fatty acids such as stearic and palmitic acids, are major constituents of sperm plasma membranes and serve as substrates for β-oxidation, contributing to both membrane fluidity and energy supply required for sperm motility and viability (Amaral, 2022; Satorre et al., 2025). Two distinct metabolic clusters were observed, indicating notable inter-individual variation despite standardized feeding and management practices. In contrast, several bulls exhibited higher levels of alternative metabolites, supporting the clustering-derived metabolomic heterogeneity and suggesting differences in energy metabolism or spermatogenic efficiency among individuals. In this context, the abundance of fatty acyls, particularly stearic and palmitic acids, was associated with differences in sperm lipid composition and potential impacts on membrane integrity and metabolic function post-thaw. Similar results have been reported in beef and dairy bulls, where fatty acid metabolism has been identified as a key determinant associated with fertility (Menezes et al., 2019; Longobardi et al., 2020). Palmitic acid, which showed the highest relative abundance in the current dataset, is particularly important due to its structural role in sperm membranes and its association with reproductive potential and semen quality (Velho et al., 2018; Longobardi et al., 2020).

The saturated fatty acids palmitic, stearic, and myristic acids showed strong positive relationships with one another, suggesting coordinated regulation. Unlike unsaturated fatty acids, saturated fatty acids increase lipid packing density and membrane rigidity; therefore, their co-occurrence may contribute to enhanced structural stability and reduced susceptibility to lipid peroxidation in sperm membranes during cryopreservation (Watson, 2000). These metabolites were consistently identified as the most discriminant substances, highlighting their crucial role in the formation of unique metabolic profiles in individual bulls. Surprisingly, serine was only found in the Rowa bull sample, indicating a metabolic response that is unique to each individual and may be connected to one-carbon metabolism or amino acid-mediated antioxidant defense (Yang & Vousden, 2016; Ugur et al., 2019). These results suggest that the observed sperm metabolomic variety mostly reflects innate physiological adaptability in energy metabolism and nutrient utilization under regulated dietary and managerial settings. The abundance and composition of key metabolites that regulate sperm and functional integrity are likely influenced by this systemic sperm metabolic response.

Under uniform feeding and management, a pattern of associations between systemic biochemistry and sperm metabolites was observed. Blood biochemistry related to lipid transport and ionic tone showed associations with intracellular lipid and energy-related metabolites in sperm, and a subset of these metabolites tracked post-thaw resilience, higher viability, greater PMI, and lower morphological abnormalities. This extends our earlier evidence of lipid-centric metabolomic separation among bulls and supports a potential link between nutritional status, metabolic profiles, and reproductive traits. Albumin was positively associated with sperm palmitic acid, consistent with its role as a major fatty acid carrier that facilitates membrane remodelling in gametes (Peris-Frau et al., 2020). Potassium displayed a bidirectional pattern, negative with citric acid and D-fructose but positive with palmitic acid, indicating that electrolyte homeostasis may be associated with variations in glycolytic/tricarboxylic acid (TCA)-related metabolites while favouring saturated fatty acid availability for membrane stabilization during cryostress (Peris-Frau et al., 2020; Yeste, 2016). Additional links were identified between total protein and palmitic acid, total protein and cholesterol (negative correlation), chloride and caproic acid, and total CO_2_ and glycerol monostearate (negative correlation). these findings indicate associations between systemic protein levels, acid–base balance, ionic status, and variations in sperm lipid and glycerolipid composition relevant to cryoresilience.

Functionally, metabolites indicative of lipid turnover and short-chain fatty acids (SCFAs) were associated with cell recovery after the thawing process. 1-monopalmitin and nonadecanoic acid correlated positively with viability, consistent with the view that glycerolipids remodelling preserves bilayer fluidity and mitigates destructive phase transitions during freezing/thawing (Peris-Frau et al., 2020; Yeste, 2016). Caproic acid, valeric acid, and 6-methyl-2-pyridinecarboxylic acid were positively correlated with %PMI. While direct SCFA signalling in bovine sperm requires targeted testing, our observations are consistent with reports that SCFAs modulate sperm signalling and migration through the olfactory GPCR OR51E2, supporting a plausible receptor-mediated mechanism by which small metabolites may influence membrane stability under cryo-ROS conditions (Teveroni et al., 2022). Conversely, dodecanoic acid and glycerol monostearate showed strong negative correlations with morphological abnormalities, suggesting that specific saturated fatty acids and glycerolipids may act as structural buffers against cryo-induced deformation, consistent with membrane-centric cryodamage frameworks (Peris-Frau et al., 2020; Yeste, 2016). No significant associations were detected between metabolites and post-thaw motility, a biologically relevant observation given the central role of motility in fertility evaluation. This suggests that motility recovery may depend on physiological processes not captured in the detected metabolite panel, particularly mitochondrial activity and ATP availability (Gonzalez et al., 2022). This interpretation is consistent with the absence of mitochondrial functional measurements in the present study, representing a key limitation.

The systemic signals, particularly lipid carriage *via* albumin and potassium-related shifts in substrate use, align with the lipid and short-chain fatty acid features in Table 5 that are associated with higher viability and greater %PMI, a pattern consistent with membrane-centric cryobiology (Peris-Frau et al., 2020; Yeste, 2016). The next steps include targeted tandem mass spectrometry to confirm the top signals (Pang et al., 2024), membrane-oriented lipidomics to resolve phospholipid and cholesterol remodelling (Peris-Frau et al., 2020), and mitochondrial function assays to address the lack of motility associations (Gonzalez et al., 2022). Evidence that short-chain fatty acids modulate sperm signalling and redox defenses (Teveroni et al., 2022) further supports these steps toward biomarker-guided sire selection and nutrition-informed reproductive management.

## Conclusions

This integrative study in *B. javanicus* maintained under standardized nutrition demonstrates a systemic link between blood biochemical status, sperm metabolomic composition, and post-thaw functional competence. By combining blood biochemistry with untargeted GC–MS of washed sperm pellets from freeze-thawed ejaculates, this study identified specific blood and sperm metabolomic parameters associated with bull sperm cryotolerance. In blood, albumin, chloride, total protein, and total CO_2_ were associated with cryoresponsive traits. In sperm, palmitic acid, stearic acid, myristic acid, caproic acid, cholesterol, and glycerol monostearate were linked to post-thaw viability, plasma membrane integrity, and morphological normality. The association between chloride and caproic acid was particularly related to membrane integrity, whereas no significant associations were observed with motility. These findings identify lipid- and electrolyte-related metabolites as candidate biomarkers requiring targeted validation. Once validated, these biomarkers may support practical sire screening and inform optimization of semen extender formulations and cryopreservation protocols in artificial insemination programs.

## Supplemental Information

10.7717/peerj.21244/supp-1 Supplemental Information 1 Raw GC–MS metabolomics data generated from Bali bull sperm samples, including the detected peaks and intensity values.

10.7717/peerj.21244/supp-2 Supplemental Information 2 ARRIVE checklist

## Additional Information and Declarations

### Data Availability

The following information was supplied regarding data availability: The raw data is available in the Supplemental File. The raw data is available at Metabolights: MTBLS13639.
